# Management of overwintering pine sawyer beetle, *Monochamus alternatus* with colonized *Beauveria bassiana* ERL836

**DOI:** 10.1371/journal.pone.0274086

**Published:** 2022-09-02

**Authors:** Jong-Cheol Kim, Mi Rong Lee, Jeong Seon Yu, So Eun Park, Panjung Ha, Jae Su Kim

**Affiliations:** 1 Department of Agricultural Biology, College of Agriculture & Life Sciences, Jeonbuk National University, Jeonju, Korea; 2 Crop Protection R&D Center, Farm Hannong (LG Affiliated Co.), Nonsan, Korea; 3 Department of Agricultural Convergence Technology, Jeonbuk National University, Jeonju, Korea; Benemérita Universidad Autónoma de Puebla: Benemerita Universidad Autonoma de Puebla, MEXICO

## Abstract

*Monochamus alternatus* is a major forest pest that spreads pine wilt disease in pine trees as a vector of pine wilt nematodes. Chemical insecticides used as fumigants to control overwintering *M*. *alternatus* in forests are highly toxic to the environment, so we investigated entomopathogenic fungus *Beauveria bassiana* ERL836 as an eco-friendly and alternative material to control overwintering *M*. *alternatus*. In this work, we evaluated the insecticidal activity of *B*. *bassiana* ERL836 against *M*. *alternatus* adults, the possibility of fungal colonization on pine tree bark, and finally the control efficacy of fungal pre-treatment on pine tree logs against emerging *M*. *alternatus* adults in semi-field and field conditions. *M*. *alternatus* adults were killed on the pine tree logs pre-treated with the *B*. *bassiana* ERL836. White conidia were observed not only on the surface of the dead adults but also on the pine tree logs, suggesting that the adults were killed by the fungus on the pine. A formulated ERL836 powder treatment on larvae-infested pine logs showed high insecticidal activity against adults, similar to that with the fungal powder suspension treatment, but we demonstrated that using the fungal powder was simpler than using the suspension in field conditions. Even in the field condition, the fungal powder treatment showed high insecticidal activity against *M*. *alternatus* adults, which we attribute to its ability to maintain fungal activity for a long time in field conditions by covering the pine tree logs with a film during overwintering. We confirmed that the risk that fungus-infected *M*. *alternatus* adults would spread the fungus to other non-target forest insects was low. Thus, even a high-concentration treatment in a specific area is unlikely to transmit the fungus outside that area, so it can be safely used to control this pine wilt nematode vector in forest ecosystems.

## Introduction

Pine wilt disease (PWD) is a serious problem in pine forests. The disease infects and damages pine trees, destroying forest ecosystems and lowering the quality of pine tree products, which causes serious economic damage [1–3]. PWD has been reported in a wide area throughout East Asia and Europe, specifically in Korea, China, Japan, and Taiwan in East Asia and in Portugal and Spain in Europe. In all those countries, PWD causes economic losses every year [4–9]. Furthermore, the spread of PWD is expected to increase as the climate changes, so the damage it causes could also increase [10]. PWD is cause by pine wilt nematodes (PWN), *Bursaphelenchus xylophilus*, which are mediated by *Monochamus* sp. [1, 2]. In particular, the Japanese pine sawyer beetle, *M*. *alternatus*, is a major pest that mediates PWN. To prevent the damage caused by PWD, strategies to manage PWN and its major vector pests, are in current use.

There are three main way to manage *M*. *alternatus*, and each of these ways has their own disadvantages. 1) The method of spraying the chemical control agents such as acetamiprid, fenitrothion, thiacloprid, thiamethoxam, and clothianidin in the *M*. *alternatus* adults activity area can control a large area, but it may have a negative effect on the environment and non-target insects by the agents used [7, 11, 12]. 2) The method of cutting down, incinerating or crushing PWD-infected pine trees requires labor and time because the pine trees cut from the forest must be moved to the outside [12]. 3) The method of fumigating chemicals by cutting down PWD-infected pine trees, piling them nearby, covering them with a tarpaulin, and uses highly concentrated chemical fumigants such as metam sodium, which can cause various diseases to humans [13–15]. The use of chemical agents is particularly limited because it affects forests that support various biota.

The demand for eco-friendly insecticidal materials using microorganisms has increased due to the environmental and human toxicity problems of chemical agents, In particular, studies on PWN-mediated pests using entomopathogenic fungi have been reported. *Beauveria* sp. [16–19] and *Metarhizium* sp. [20–22] were reported to have high virulence against *Monochamus* sp., which are PWN vectors. The control efficacy of those fungi against *Monochamus* sp. has been studied using fungal nonwoven fabric, fungal pellets, pine bark colonization isolates, and fungal spray [17, 20, 23–26]. We previously studied *M*. *anisopliae* as a fumigant substitute and confirmed its feasibility by examining its control efficacy [20, 26]. However, we considered that it was necessary to evaluate the stability and efficacy of formulated fungal material and the effect on the non-target insects by the treated fungus.

In this study, we tested a method for managing the PWN-mediate *M*. *alternatus* using the commercially available entomopathogenic fungus *B*. *bassana* ERL836 from our previous works, an eco-friendly microbial material. To evaluate the practicality of expanding application of the fungus to control thrips, we tested the insecticidal activity of ERL836 against *M*. *alternatus*, the fungal colonization on pine bark, and the control efficacy of formulated ERL836 powder in tree logs artificially infested with *M*. *alternatus* in various settings and finally in fields. We also evaluated the insecticidal activity of the fungus against non-target insects and the possibility that *M*. *alternatus* infected with *B*. *bassiana* ERL836 could transmit the fungus. Based on the results of comprehensive our study, we suggest an eco-friendly management factor that can protect the pine forest from PWD.

## Materials and methods

### Fungi and insects

*B*. *bassiana* ERL836 (KCCM11506P) was provided by the Entomology Research Laboratory (University of Vermont, Burlington, VT, USA), and the *B*. *bassiana* ERL836-egfp transformant was obtained using the *Agrobacterium tumefaciens*-mediated transformation method [27]. The strains were stored at the Insect Microbiology & Biotechnology Laboratory (Jeonbuk National University, Jeonju, Korea) as conidial suspensions in 20% glycerol at -80°C (glycerin, Daejung, Korea) until use [28]. The strain was cultured on quarter-strength Sabouraud dextrose agar medium (1/4 SDA, BD Difco^TM^, Sparks, MD, USA) in Petri dishes (dia. 60 mm) at 27°C for 14 days to induce conidial production. The conidia of the strain were harvested by putting the agar blocks in which the strain was cultured in a 15-ml conical tube (SPL Life Science Co., Pocheon, Korea) with 5 ml of 0.03% siloxane solution (Silwet, FarmHannong Inc., Nonsan, Korea) and creating a suspension using a vortex mixer (WiseMix VM-10, Daihan, Korea). The numbers of conidia were counted three times at 400× magnification using a hemocytometer. The conidia suspension was diluted to 1.0 × 10^7^ conidia/ml with 0.03% siloxane solution. Then, 2 μg of the conidia suspension was inoculated in 1/4 SDA medium at 27°C for 24 h, and a conidia germination rate of >90% was confirmed.

The experimental insects (*Monochamus alternatus*, *Bombyx mori*, *Allomyrina dichotoma*, and *Lucanus maculifemoratus*) were supplied by OsangKinsect (Yesan, Korea), an insect rearing company in Korea. All the insects were stored in insect breeding dishes at 25 ± 2°C. The 4^th^ instars of *B*. *mori* larvae were used, and the adults of the others insects were used within ten days of emergence. Additionally, for the indoor and semi-field experiments with wintering pine trees, holders were drilled into a pine tree, and 5^th^ instar *M*. *alternatus* larvae were artificially inserted.

### Insecticidal efficacy test of ERL836 against adults on pine tree in pot condition

The insecticidal activity of *B*. *bassiana* ERL836 against *M*. *alternatus* adults was evaluated by pre-treating pine trees (*Pinus densiflora*) with a fungal conidia suspension (1.0 ×10^6^ or 1.0 ×10^7^ conidia/ml) in pot conditions and then adding *M*. *alternatus* adults to the trees. Four pine trees with a height 80 cm were planted in a plastic pot (40 × 30 × 30 cm) with soil, and adequate water was supplied. The pine trees in pots were maintained for 7 days at 25 ± 2°C, relative humidity (RH) 60 ± 10%, and photoperiod L:D = 16:8 h in laboratory conditions to acclimatize. Each pot was then covered with a 200 mesh net (40 × 30 × 100 cm), and ten *M*. *alternatus* adults were placed inside the net. The numbers of dead and surviving adults were counted daily for 25 days (25 ± 2°C, RH 70 ± 10%, photoperiod L:D = 16:8 in laboratory conditions), and mycosis was observed. As a non-treated control, a pine tree was sprayed with 100 ml of 0.03% siloxane solution. All treatments were tested in triplicates.

### Insecticidal efficacy test of ERL836 against adults using artificially larvae-infested pine tree logs

Fifth instar *M*. *alternatus* larvae were inserted into pine logs (length 30 cm and 20 cm diameter) using 100 mm long holes that were then blocked using a piece of pine wood. Five holes were drilled per log, and one larva was placed in each hole. Then, 100 ml of *B*. *bassiana* ERL836 conidia suspension (1.0 × 10^7^ conidia/ml) diluted with 0.03% siloxane solution was sprayed onto the entire log surface and dried at room temperature for 24 h. In a plastic container (30×40×30 cm^3^), 300 g of soil was laid on the floor of the container, and 100 ml of distilled water was sprayed onto the entire soil surface. Two pine logs were placed in each plastic container, which was then sealed with a lid. The emerging adults and dead and alive adults were counted daily for 25 days (25 ± 2°C, RH 95 ± 5%, L:D = 16:8). As a non-treated control, a pine log was treated with 0.03% siloxane solution. All treatments were tested in triplicates.

Secondly, insecticidal activity against *M*. *alternatus* was evaluated using a fungal powder formulation. The insecticidal activity of *B*. *bassiana* ERL836 against *M*. *alternatus* was evaluated in the form of a 2.5% powder formulation which was made by FarmHannong (Nonsan, Korea). The *B*. *bassiana* ERL836 powder formulation was diluted in water for spray or directly powered to pine logs. For the powder-based suspension spray, 1 and 10 g of fungal powder formulation were diluted with 100 ml water to prepare 1% and 10% suspensions, respectively. For the powdering of the formulation, 1 g and 10 g of fungal powders were scattered onto the pine logs in powder form. Plastic boxes containing pine logs artificially infected with *M*. *alternatus* larvae were prepared as described above. The entire surface of each pine log was treated with *B*. *bassiana* ERL836 according to the fungal powder treatment type. Emerging adults and dead and alive adults were counted daily for 40 days after the emergence of the first adults. All treatments were tested in triplicate in greenhouse conditions. The temperature and relative humidity were recorded every 1 hour with a HOBO U12-012 External Temperature/Relative Humidity/Light/External data logger (Onset Computer Co., Bourne, MA, USA).

### Colonization of *B*. *bassiana* ERL836 on pine bark

Before we applied the *B*. *bassiana* ERL836 in the field, we assessed the ability of the fungus to colonize pine bark. Three pine barks (*Pinus densiflora*, *P*. *koraiensis* and *P*. *thunbergia*) were collected from trees growing wild in a forest. They were cut into discs using a cork borer (dia. 6 mm) and autoclaved at 121°C for 15 min. A filter paper (dia. 55 mm) was placed in a Petri dish (60 × 15 mm), and a 40 × 40 mm piece of Parafilm (Heathrow Scientific, Vernon Hills, IL, USA) was placed on the filter paper to maintain high humidity. The nine pieces of bark were placed on the Parafilm at 10-mm intervals, and conidia suspensions (1.0 × 10^7^ conidia/ml) of *B*. *bassiana* ERL836 and ERL836-egfp were inoculated at 20 μl per piece. The Petri dish was kept at room temperature for 24 h without a lid so that the conidia suspensions on the bark could dry naturally. Then 400 μl of distilled water was placed on the filter paper, and the Petri dish was closed and sealed with Parafilm. All treatments were maintained at 27°C for 14 days, and then the growing fungus was observed with a microscope at × 10 magnification. The fluorescence expression of the egfp was observed with a fluorescence stereo microscope (SMZ1270, Nikon Co., Tokyo, Japan) to confirm that the growth was *B*. *bassiana* ERL836. The negative control bark was treated with 0.03% siloxane solution. All treatments were assayed in triplicates.

### Semi-field test using wintering pine logs artificially infested with larvae

The semi-field test was performed from spring to fall (April to July 2020) at FarmHannong’s test field (36°07’58.1"N 127°07’45.4"E, Nosan, Korea). Pine logs (ca. 1 m) with five final-stage larvae of *M*. *alternatus* were supplied by the insect rearing company OsangKinsect, Korea. In the test field, an iron frame (3×4×4 m) was installed, and the entire frame was covered with a black film to create shade. We treated twelve pine logs (about 60 *M*. *alternatus* larvae) with 100 g of 2.5% formulated *B*. *bassiana* ERL836 fungal powder formulation, and then the logs were piled to about 1 m^3^ in the frame. The fungal-treated pine logs were covered with a covering film (tarpaulin) and sealed. Each treatment was tested in triplicate (**S1A Fig**). The number of emerging adults and mortality of *M*. *alternatus* adults were monitored weekly from June to July 2020. Field verified mortality in each monitoring time was pooled. Alive emerging adults were moved to a lab and kept at a moisturising Petri-dish for 14 days to assess potential infection and mortality (this data was also pooled). The temperature and relative humidity were recorded every 1 hour with a HOBO U12-012 External Temperature/Relative Humidity/Light/External data logger (Onset Computer Co., Bourne, MA, USA). All treatments were tested in triplicates. The mortality was investigated on the day of collection and after moist treatment for 14 days after collection, and the number of alive and dead adults was calculated cumulatively for individuals collected for 6 weeks. The mortality was calculated as the ratio of the cumulative number of alive and dead adults at each time point.

### Field test

The field test was performed from fall to summer (November 2019 to July 2020) in a forest area where pine wilt nematode occurred (35°29’52.0"N 129°17’35.8"E, Ulsan, Korea). Near that forest area, pine logs (ca. 1 m) naturally infested with *M*. *alternatus* larvae were collected with the assistance of a local forest manager during the winter (November to December 2019). The 2.5% formulation of *B*. *bassiana* ERL836 fungal powder was treated to about 50 pine logs presumed to contain about 200 *M*. *alternatus* larvae, piled to about 1 m^3^, and covered with a covering film (tarpaulin), and sealed. To evaluate the insecticidal activity according to the amount of fungal powder formulation, 100 g and 200 g of the *B*. *bassiana* ERL836 fungal powders were treated, and the non-treated control was not treated with any fungal powder. All treatments were tested in triplicate (**S1B Fig**). The number of emerging adults and the mortality of *M*. *alternatus* adults were monitored weekly. Field verified mortality in each monitoring time from Jun 17th to August 5th was pooled. Alive emerging adults were moved to a lab and kept at a moisturising Petri-dish for 14 days to assess potential infection and mortality, and the data was also pooled. The mortality was investigated on the day of collection and after moist treatment for 14 days after collection, and the number of alive and dead adults was calculated cumulatively for individuals collected for 8 weeks. The mortality was calculated as the ratio of the cumulative number of alive and dead adults at each time point.

To confirm the *B*. *bassiana*-mediated mortality, fungal colonies were isolated from the cadavers of *M*. *alternatus* adults and cultured in 1/4 SDA medium with 100 μg/ml of dodine (Sigma-Aldrich, Saint Louis, MO, USA), 100 μg/ml of streptomycin (Sigma-Aldrich, Saint Louis, MO, USA), and 200 μg/ml of chloramphenicol (Sigma-Aldrich, Saint Louis, MO, USA) [29]. The DNA of the fungus was extracted using a DNA extraction kit and identified by sequencing the ITS region. The full ITS region was amplified using the universal primer set ITS1F (5′‐CTT GGT CAT TTA GAG GAA GTA A‐3′) and ITS4R (5′‐TCC TCC GCT TAT TGA TAT GC‐3′) [30]. The temperature and relative humidity were recorded every 1 hour with a HOBO U12-012 External Temperature/Relative Humidity/Light/External data logger (Onset Computer Co., Bourne, MA, USA). All treatments were tested in triplicates.

### Transmission of *B*. *bassiana* ERL836 to non-target insects

First, the direct insecticidal activity of *B*. *bassiana* ERL836 against the three non-target forest insects was investigated. The three non-target insects were *A*. *dichotoma* adult, *L*. *maculifemoratus* adult, and *B*. *mori* larvae. Each insect was placed in a plastic cup with a filter paper soaked in distilled water on the bottom of the cup, and then 1 ml of the fungal conidial suspension (1.0 × 10^7^ conidia/ml) was adjusted with 0.03% siloxane solution was sprayed onto the insects. A food source was provided to each insect, and the lid of the cup was closed. The dead and alive insects were counted daily for 14 days (25 ± 2°C). All treatments were tested in three replicates of ten insects each.

The transmission potential of *B*. *bassiana* ERL836 to the non-target forest insects was confirmed using fungus-infected *M*. *alternatus* adults. A 1-ml conidia suspension of *B*. *bassiana* ERL836 (1.0 × 10^7^ conidia/ml) adjusted with 0.03% siloxane solution was sprayed onto a male adult *M*. *alternatus* and transferred to a plastic cup (lid 10 cm, bottom 60 cm, height 15 cm). The plastic cup was layered with a filter paper (dia. 55 mm), moisturized with 1 ml of distilled water. *A*. *dichotoma* adult, *L*. *maculifemoratus* adult, or *B*. *mori* larva was placed in each cup where a fungus-infected *M*. *alternatus* male adult was previously placed (one infected *M*. *alternatus* + one non-target insect per plastic cup). A pine branch (ca. 150 mm) was provided as food, and the lid was closed and sealed. The number of dead and alive adults was counted daily for 14 days (25 ± 2°C). As a negative control, a *M*. *alternatus* male adult was treated with 0.03% siloxane solution, placed in the plastic cup with each insect, and the lid was closed. All treatments were tested in three replicates of ten adults each.

### Statistical analysis

All data on the mortality of *M*. *alternatus* adults, the control efficacy, and mortality of the target and non-target insects were arc-sine transformed after an analysis of the normal distribution and then analyzed using a generalized linear model and Tukey’s honestly significant difference test for multiple comparisons. All analyses were performed in SPSS ver. 22.0 (IBM Corp., Armonk, NY, USA) at the 0.05 (α) level of significance.

## Results

### Insecticidal activity of *B*. *bassiana* ERL836 against *M*. *alternatus* adults under pot conditions

**Fig 1:**
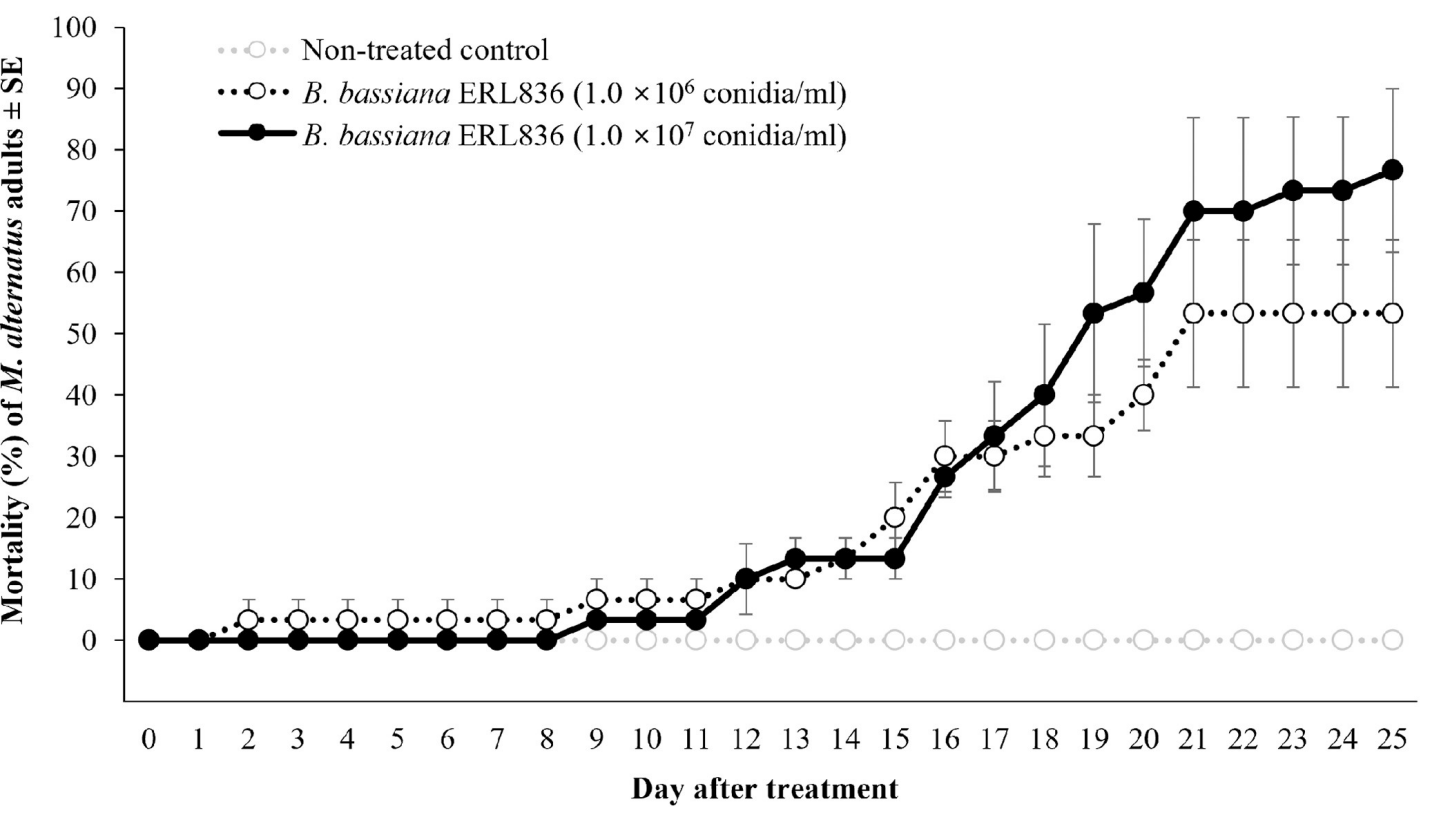
Mortality of *M*. *alternatus* adults in small pine trees pre–treated with *B*. *bassiana* ERL836 in pot conditions. *B*. *bassiana* ERL836 conidia suspension (1.0 × 10^6^ or 1.0 × 10^7^ conidia/ml) was treated on four pine trees in a pot (40 × 30 × 30 cm) and ten *M*. *alternatus* adults were released into the pot (10 adults/pot). The numbers of alive and dead adults were counted daily.

In the small pot of pine tree condition, *M*. *alternatus* adults exposed to pine trees pre-treated with *B*. *bassiana* ERL836 died on nine days after the release of adults (**Fig 1**). The mortality of *M*. *alternatus* adults was 53.3 ± 12.0% in the 1.0 × 10^6^ conidia/ml treatment and 76.7 ± 13.3% in 1.0 × 10^7^ conidia/ml treatment on 25 days after release of adults. In the non-treated group, there were no dead adults on 25 days after release. Thus, the mortality of *M*. *alternatus* adults was significantly increased by the fungal pre-treatment, and it increased dose-dependently with the fungal conidia concentration (*F*_*2*,*156*_ = 224.4, *p* < 0.001). In the fungal treatment groups, white conidia covered the surface of dead *M*. *alternatus* adults.

### Insecticidal activity of pre-treated *B*. *bassiana* ERL836 on pine logs artificially infected with larvae

**Fig 2:**
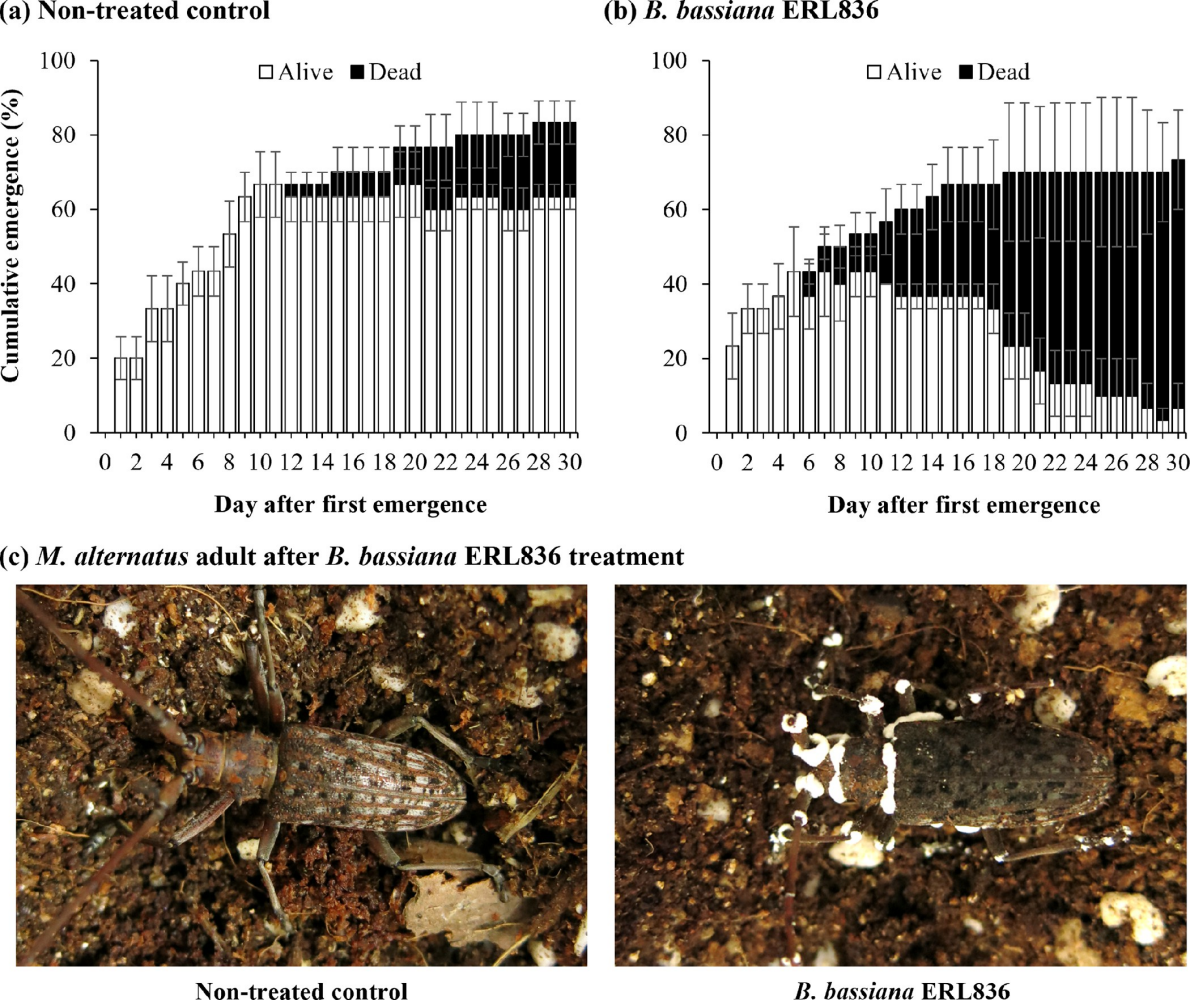
Insecticidal activity of *B*. *bassiana* ERL836 against *M*. *alternatus* adults using fungal pre–treatment of pine logs artificially infested with larvae. The 100–ml fungal conidia suspension (1.0 × 10^7^ conidia/ml) was sprayed onto pine logs artificially infested with 5th instar larvae of *M*. *alternatus*, and then the logs were placed in a plastic box, and the lid was closed and sealed. Cumulative emergence of *M*. *alternatus* adults in the non–treated control (a) or ERL836 treatment (b) was counted daily after the first observation of emergence. In the emergence, the percentages of alive and dead numbers were presented as white and black, respectively. A mycotized adult was observed in the ERL836 treatment (c).

When we infested pine logs with *M*. *alternatus* larvae in indoor conditions and then treated the logs with *B*. *bassiana* ERL836, we found that *M*. *alternatus* adults began to emerge on the 30th day (**Fig 2**). The emergence rate of adults was counted beginning when the first adult emerged from the pine logs. The non-treated logs and *B*. *bassiana* ERL836–treated logs showed the emergence of 20.0 ± 5.8% and 23.3 ± 8.8%, respectively, on the first day and 83.3 ± 6.7% and 73.3 ± 6.7%, respectively, on the 30th day. In the non-treated group, the first adults died on 12 days after emergence, and the mortality of adults was 20.0 ± 5.8% on the 30th day. In the *B*. *bassiana* ERL836 treatment, the first adults died on the 6th day after emergence, and the mortality rate was 66.7 ± 13.3% on the 30th day. Thus, the emergence-based mortality was 88.9 ± 11.1% in the *B*. *bassiana* ERL836 treatment and 23.3 ± 5.5% in the non-treated control (**Fig 2A** and **2B**), which was a significant difference (*F*_*1*,*124*_ = 164.4, *p* < 0.001). The growth of mycelium and white conidia was observed mainly on the segment parts of the cadavers in the ERL836 treatment, which indicates that the *M*. *alternatus* adults died from exposure to the *B*. *bassiana* ERL836 (**Fig 2C**).

**Fig 3:**
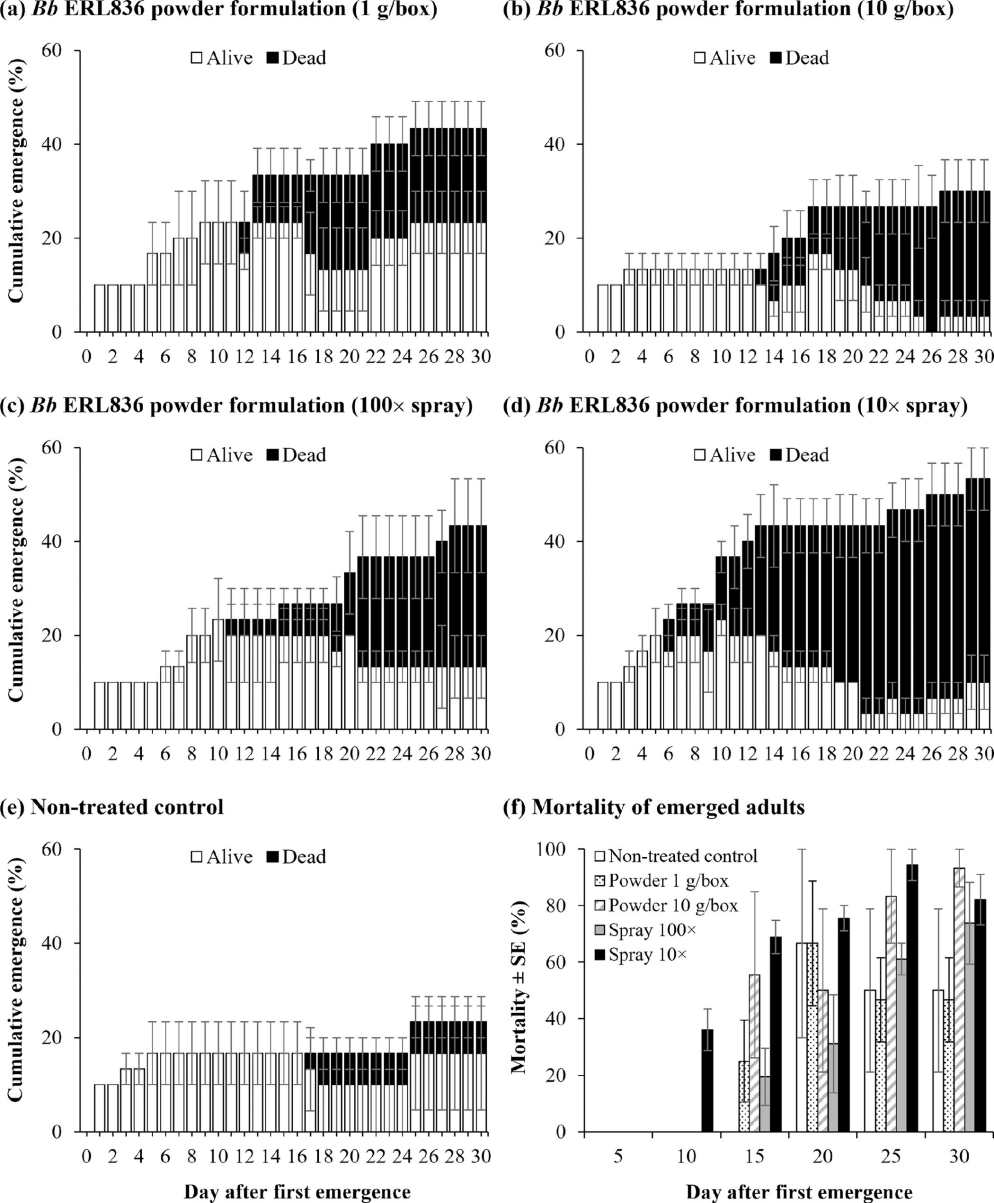
Insecticidal activity of *B*. *bassiana* ERL836 against *M*. *alternatus* adults using formulated fungal powder pre–treatment of pine logs artificially infested with larvae in greenhouse conditions. The fungal powder formulation was directly powdered on the logs at 1g/box (a) and 10 g/box (b). Secondly the fungal powder formulation was diluted with water at 100× (c) and 10× (d) and sprayed on the logs. Non–treated control was designed (e). The cumulative emergence of *M*. *alternatus* adults was counted daily after the first observation of emergence. In the emergence, the percentages of alive and dead numbers were presented as white and black, respectively. The final mortality of each treatment was summarized (f).

When we artificially infested pine logs with *M*. *alternatus* larvae and then treated the logs with the fungus *B*. *bassiana* ERL836 formulated as powder in greenhouse conditions, the first adults emerged from the pine logs about 90 days after the fungal treatment (**Fig 3**). The average temperature in the greenhouse was 16.3 ± 3.0°C (range 8.5–28.2°C), the relative humidity was maintained above 90%, and dark conditions were maintained. In the group treated with 1 g/box of fungal powder, the first adults died on 12 days after their first emergence, and the emergence and mortality rates on the 30th day were 43.3 ± 3.3% and 20.0 ± 5.8%, respectively (**Fig 3A**). In the 10 g/box fungal powder treatment, the first adults died on 13 days after their emergence, and the emergence and mortality rates on the 30th day were 30.0 ± 10.0% and 26.7 ± 6.7%, respectively (**Fig 3B**). In the 100× fungal suspension spray using the formulated powder, the first adults died on the 12th day after their emergence, and the emergence and mortality rates on the 30th day were 43.3 ± 14.5% and 30.0 ± 10.0%, respectively (**Fig 3C**). In the 10× fungal suspension spray using the powder, the first adults died on the 6th day after their emergence, and the emergence and mortality rates on the 30th day were 53.3 ± 8.8% and 43.3 ± 6.7%, respectively (**Fig 3D**). In the non-treated control, the first adults died on 17 days after their emergence, and the emergence and mortality rates on the 30th day were 23.3 ± 8.8% and 6.7 ± 3.3%, respectively (**Fig 3E**). There was no significant difference between the 10 g/box powder treatment and the non-treated control (*p*>0.05), and the 10× suspension spray showed the highest emergence rate (*F*_*4*,*310*_ = 36.6, *p* < 0.001). When we calculated the ratio of dead adults/total emerged adults on the 15th and 30th days after the first emergence of adults, the non-treated control was 0.0% and 50.0 ± 28.9%, and the other treatments were as follows (1 g/box fungal powder treatment: 25.0 ± 14.4% and 46.7 ± 14.8%, 10 g/box fungal powder treatment: 55.6 ± 29.4% and 93.3 ± 6.67%, 100× fungal suspension spray treatment: 19.4 ± 10.0% and 73.8 ± 14.5%, and 10× fungal suspension spray treatment: 68.9 ± 5.9% and 82.1 ± 9.0%, respectively) (**Fig 3F**). There was no significant difference between the 1 g/box fungal powder, 100× suspension spray, and the non-treated control. The 10 g/box fungal powder and 10× suspension spray showed significantly high insecticidal activity (*F*_*4*,*310*_ = 18.3, *p* < 0.001).

### Fungal colonization on pine bark and *M*. *alternatus* adults

**Fig 4:**
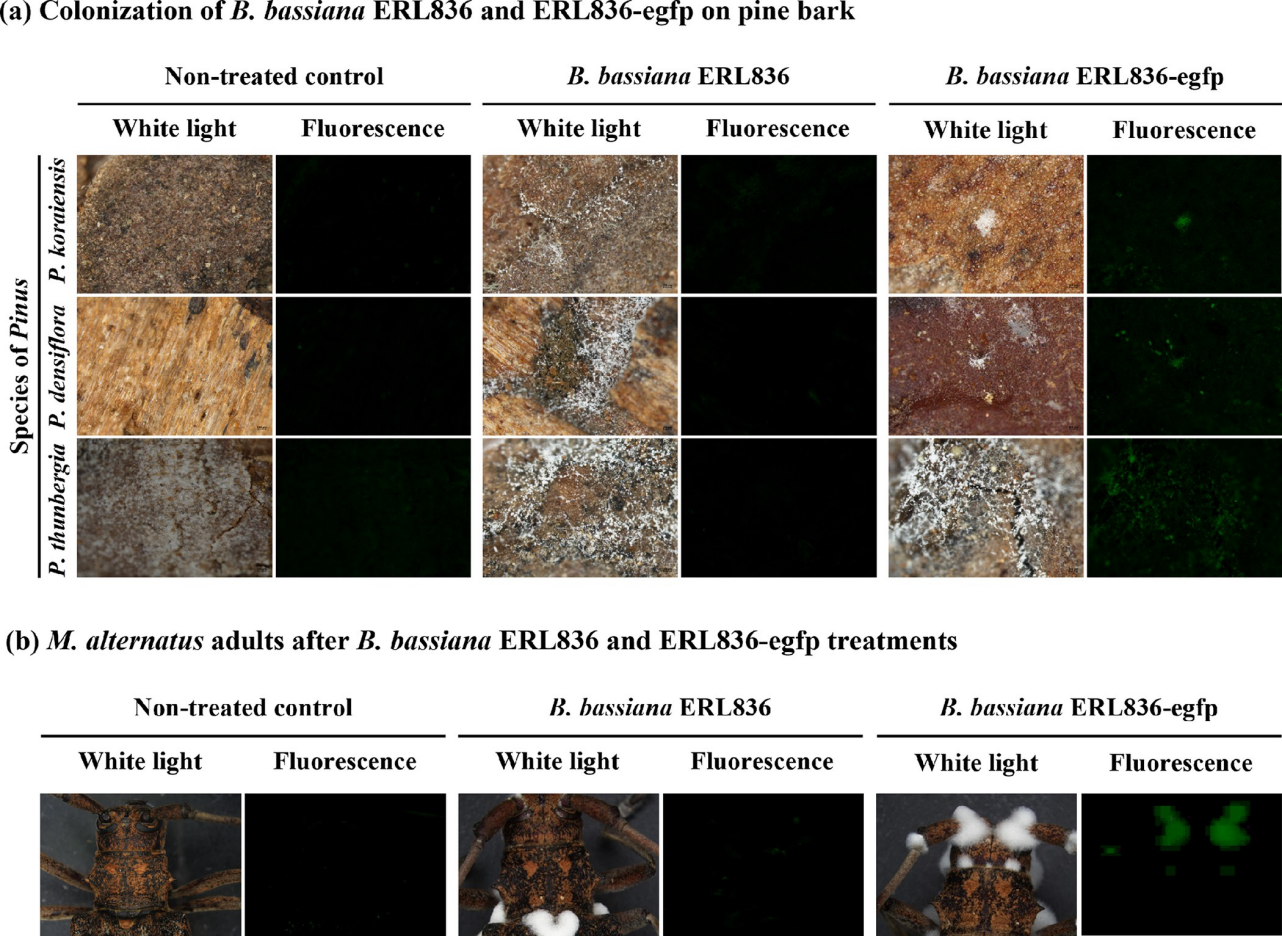
Colonization of *B*. *bassiana* ERL836 on pine tree barks. ERL836 or egfp–expressing ERL836 conidial suspension was sprayed on three different species of pine tree barks and the egfp signal was detected under a fluorescence microscope (a). The fungus–infected *M*. *alternatus* adults were observed under the same conditions at 12 days after treatment (b).

When we inoculated the sterilized bark of three pine tree species, black pine (*Pinus thunbergia*), red pine (*P*. *densiflora*), and white pine (*P*. *koraiensis*) with *B*. *bassiana* ERL836 and *B*. *bassiana* ERL836-egfp, the fungus colonized on the park and conidia were produced on the bark of all three pine species (**Fig 4A**). To confirm that the treated *B*. *bassiana* ERL836 was growing on the bark, we inoculated *B*. *bassiana* ERL836-egfp and observed fluorescence from the fluorescent strain in the areas where the hyphae and conidia were produced. The observation of egfp fluorescence indicated that the fungus growing on the bark was *B*. *bassiana* ERL836. Thus, the *B*. *bassiana* ERL836 can colonize the bark of pine trees for mycelial growth and conidia production, suggesting that emerging *M*. *alternatus* adults from the trees could come into contact with the conidia of the fungus on the barks. On the 10th day after inoculating *B*. *bassiana* ERL836 and ERL836-egfp, mycelium and white conidia were observed in the segments of *M*. *alternatus* adults (**Fig 4B**). In the adults treated with *B*. *bassiana* ERL836-egfp, fluorescence was observed where the mycelium and white conidia could be seen, suggesting that *B*. *bassiana* ERL836 killed the *M*. *alternatus* adults. In the non-treated control, *M*. *alternatus* adults did not die, and neither the conidial growth nor fluorescence of the strain was observed.

### Control efficacy of *B*. *bassiana* ERL836 in semi-field and field conditions

**Fig 5:**
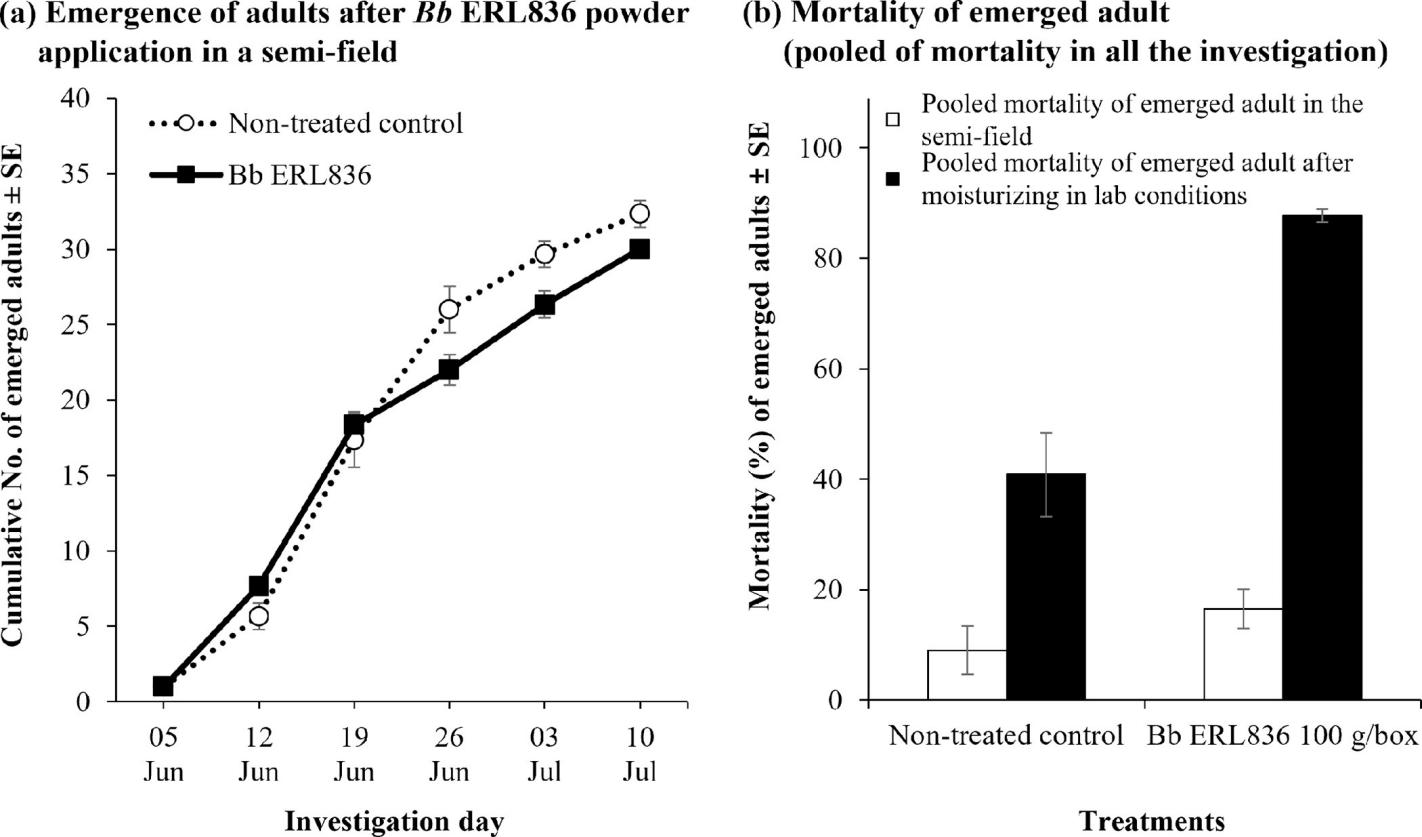
Control efficacy of *B*. *bassiana* ERL836 against *M*. *alternatus* by fungal powder formulation pre–treatment of pine logs artificially infested with larvae in semi–field conditions. The surfaces of pine logs artificially infested with *M*. *alternatus* larvae were powdered with *B*. *bassiana* ERL836 fungal powder formulation, and then the logs were piled and sealed with a film. At weekly intervals, the number of emerged and dead *M*. *alternatus* adults was counted. The error bars represent the standard error of the mean of the number of emerged adults (a). In the semi–field, mortality of adults at each investigation time was pooled, and additionally to see the possibility of infection in the alive adults, alive adults were moved to a lab and kept in moisturized Petri–dishes for 14 days and mortality was checked and pooled. The error bars represent the standard error of the mean mortality rate (b).

The control efficacy from treating larvae-infested pine logs with the formulated *B*. *bassiana* ERL836 fungal powder was evaluated in semi-field conditions (**Fig 5**). From June 5th to July 10th, the cumulative numbers of adults that emerged from the logs of non-treated control and *B*. *bassiana* ERL836 fungal powder formulation 100 g/m^3^ treatments were 32.3 ± 0.9 and 30.0 ± 0.6, respectively (**Fig 5A**). The weekly average numbers of adults emerging in the non-treated control and fungal powder formulation treatment were 5.4 ± 1.6 and 5.0 ± 1.4, respectively. The pooled mortalities of the adults on the investigation day in the non-treated control and fungal powder formulation were 9.0 ± 4.4% and 16.5 ± 3.5%, respectively (**Fig 5B**). Alive adults from the non-treated control and fungal powder treatment were collected and transferred to a lab for keeping them in a cup with high humidity for 14 days; their pooled mortalities were 40.8 ± 7.5% (non-treated control) and 87.8 ± 1.1% (ERL836 powder formulation). In the fungal powder treatment, white conidia were observed on the dead *M*. *alternatus* adults. The mortalities of adults in the non-treated control and the fungal powder treatment did not differ significantly on the investigation day, but there was a significant difference after 14 days of additional incubation under high humidity (*F*_*3*,*8*_ = 47.5, *p* < 0.001).

**Fig 6:**
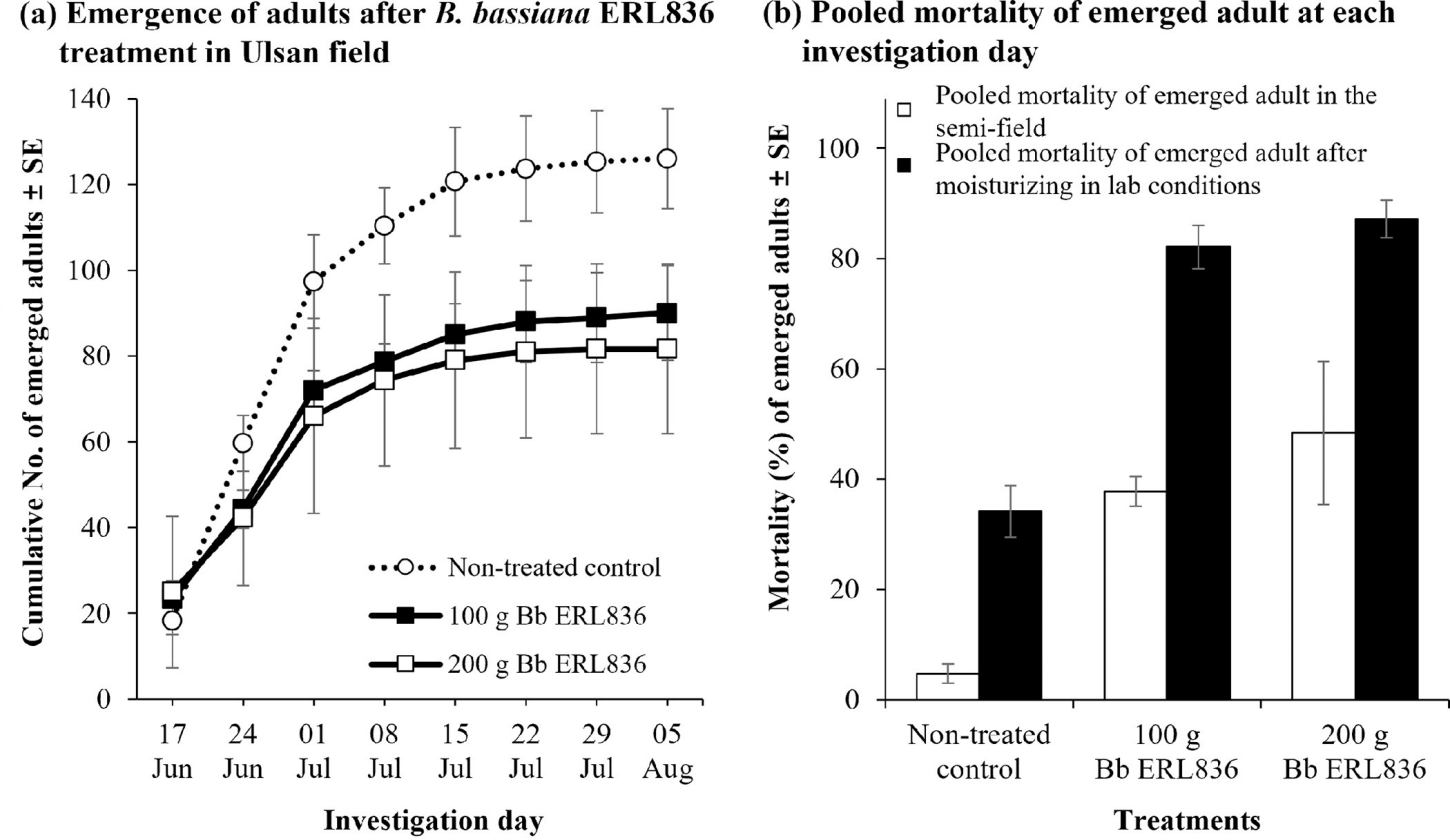
Control efficacy of *B*. *bassiana* ERL836 against *M*. *alternatus* by fungal powder formulation pre–treatment of naturally larvae–infested pine logs in forest field conditions. The surfaces of pine logs naturally infested with *M*. *alternatus* larvae were powdered with *B*. *bassiana* ERL836 fungal powder formulation, and then the logs were piled and sealed with a film. At weekly intervals, the number of emerged and dead *M*. *alternatus* adults was counted. The error bars represent the standard error of the mean of the number of emerged adults (a). In the field, mortality of adults at each investigation time was pooled, and additionally to see the possibility of infection in the alive adults, alive adults were moved to a lab and kept in moisturized Petri–dishes for 14 days and mortality was checked and pooled. The error bars represent the standard error of the mean of mortality (b).

We also tested the control efficacy of naturally infested pine logs with *B*. *bassiana* ERL836 fungal powder formulation in field conditions (**Fig 6**). From June 17th to August 10th, the cumulative numbers of adults that emerged in the non-treated control and *B*. *bassiana* ERL836 fungal powder 100 g/m^3^ and 200 g/m^3^ treatments were 126.0 ± 11.7, 90.0 ± 11.0, and 81.7 ± 19.8, respectively, and the weekly average numbers of emerged adults in each treatment group were 15.8 ± 5.6, 11.3 ± 3.9, and 10.2 ± 3.7, respectively (**Fig 6A**). The pooled mortalities of adults on the day of investigation in the non-treated control and *B*. *bassiana* ERL836 fungal powder 100 g/m^3^ and 200 g/m^3^ treatments were 4.7 ± 1.7%, 37.8 ± 2.7%, and 48.4 ± 13.0%, respectively (**Fig 6B**). When the adults were collected and kept under high humidity for 14 days, their pooled mortalities were 34.1 ± 4.7%, 82.1 ± 3.9%, and 87.2 ± 3.3%, respectively. In the fungal treatments, white conidia were observed on the dead *M*. *alternatus* adults. The pooled mortality of the adults on the day of the investigation was significantly higher in both fungal powder formulations of 100 g/m^3^ and 200 g/m^3^ (*F*_*2*,*6*_ = 13.0, *p* < 0.01). The pooled mortality of the collected adults kept in the high humidity condition confirmed that all of the fungal powder formulations had significantly high insecticidal activity (*F*_*2*,*6*_ = 39.8, *p* < 0.001). Our DNA sequencing results of the fungus collected from the infected *M*. *alternatus* adults and pine bark identified it as a *B*. *bassiana* isolate. Thus, *B*. *bassiana* ERL836 pre-treated on the pine bark grew and generated conidia that then killed the *M*. *alternatus* adults.

### Transmission of ERL836 to non-target forest insects

**Fig 7:**
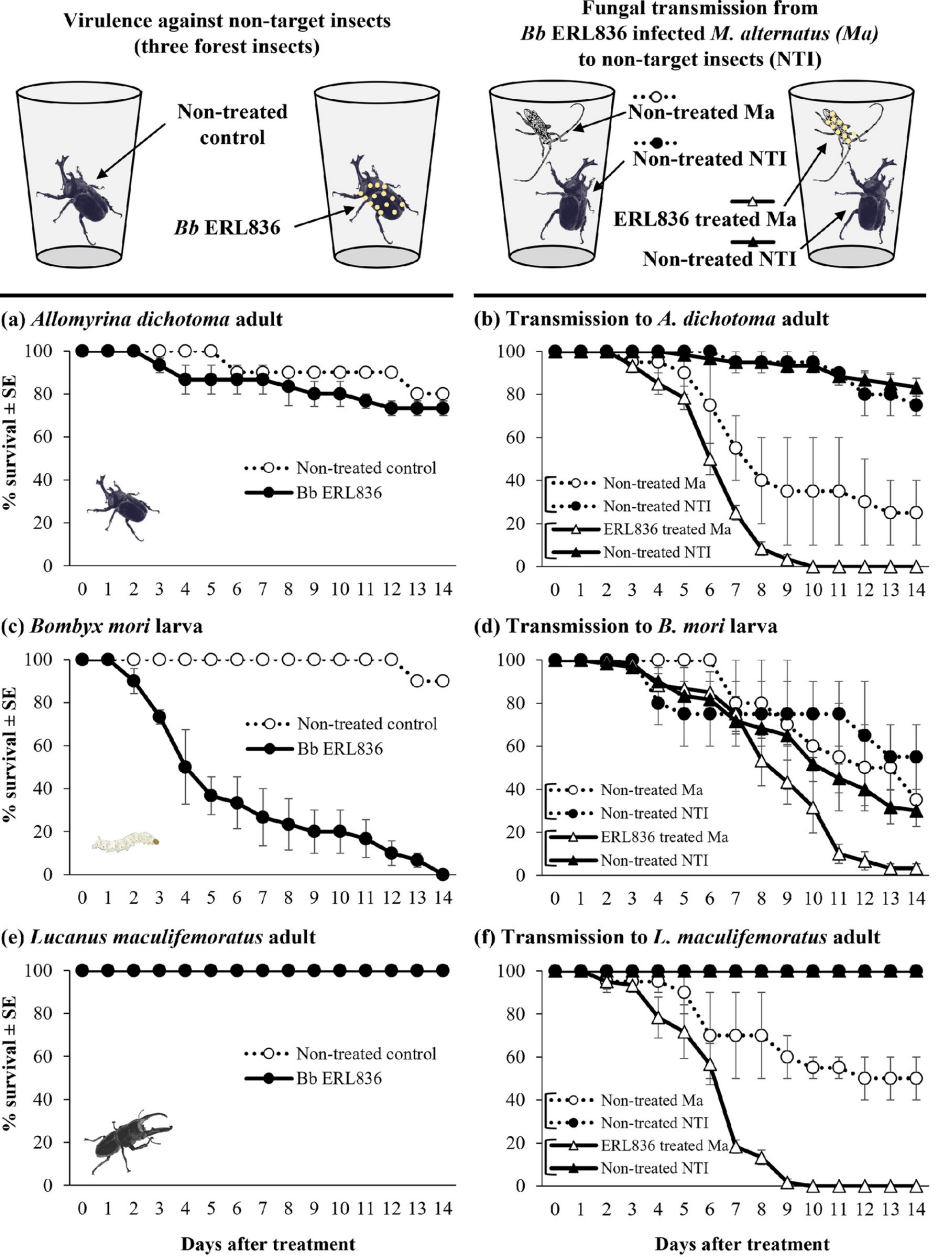
Transmission of *B*. *bassiana* ERL836 to non–target forest insects. To investigate the direct insecticidal activity, ERL836 conidia suspension at 1.0 × 10^7^ conidia/ml was sprayed on three different non–target forest insects, *A*. *dichotoma* adults (a), *B*. *mori* larvae (c), and *L*. *maculifemoratus* adults (e) in plastic container conditions. Next to see the fungal transmission from infected *M*. *alternatus* adults to non–target insects, ERL836–sprayed *M*. *alternatus* adult was kept with a non–target insect, *A*. *dichotoma* adult (b), *B*. *mori* larvae (d), and *L*. *maculifemoratus* adults (f) in the same plastic container. Non–treated *M*. *alternatus* adult was kept with the same non–target insect as a control. Percent survival was analyzed daily for 14 days.

The insecticidal activity of direct exposure to *B*. *bassiana* ERL836 and of *M*. *alternatus* adults treated with a *B*. *bassiana* ERL836 conidia suspension was evaluated on three species of forest insects (**Fig 7**). The *B*. *bassiana* ERL836-treated *A*. *dichotoma* adults had a 73.3% survival in 14 days after treatment, and the non-treated control had a 80.0% survival rate (**Fig 7A**). The *A*. *dichotoma* adults kept in the same cup as a fungus-treated *M*. *alternatus* adult had a survival of 83.3 ± 4.2% in 14 days contact, and the survival of the *A*. *dichotoma* adults kept in the same cup as non-treated control *M*. *alternatus* adults was 75.0 ± 5.0% (**Fig 7B**). The difference in the survival of *A*. *dichotoma* adults between the direct fungal exposure and infected *M*. *alternatus* exposure was not significant (*F*_*3*,*156*_ = 271.7, *p* < 0.001). The survival rate of *B*. *mori* larvae treated with *B*. *bassiana* ERL836 was 0.0% in 14 days after treatment, and the survival of the non-treated control was 90.0% (**Fig 7C**). The survival of *B*. *mori* larvae kept in the same cup as a fungus-treated *M*. *alternatus* adult was 30.0 ± 7.3% in 14 days after exposure, whereas the survival of *B*. *mori* larvae kept in the same cup as non-treated *M*. *alternatus* adults was 55.0 ± 15.0% (**Fig 7D**). The difference in the survival of *B*. *mori* larvae between the two different fungal exposures did not differ significantly, and the survival of the fungus-treated *M*. *alternatus* adults did differ significantly from that of non-treated *M*. *alternatus* adults and *B*. *mori* larvae (*F*_*3*,*156*_ = 9.8, *p* < 0.001). All the *L*. *maculifemoratus* adults in both the non-treated control and fungal treatment were alive in 14 days after treatment (**Fig 7E**), and they also survived when exposed to the fungus-treated *M*. *alternatus* adults (**Fig 7F**). The survival of *L*. *maculifemoratus* adults was not significantly affected by exposure to *M*. *alternatus* regardless of fungal treatment, and the survival of *M*. *alternatus* adults did differ significantly according to the fungal treatment (*F*_*3*,*156*_ = 465.8, *p* < 0.001).

## Discussion

In this study, we tested the potential of a commercialized entomopathogenic fungus strain, *B*. *bassiana* ERL836, as an agent for controlling the pine wilt nematode vector, *M*. *alternatus*. We devised a control strategy in which we treated the surface of pine logs where *M*. *alternatus* overwinters with the fungus instead of fumigating them with chemical agents. Then we evaluated the insecticidal activity of *B*. *bassiana* ERL836 on the emerging adults of *M*. *alternatus*. At other stages including egg, larva and pupa, *M*. *alternatus* lives inside the pine tree, which makes it difficult to evaluate the efficacy of applying fungal material to the bark. In fact, it is unreasonable to expect high insecticidal activity because this entomopathogenic fungi do not penetrate the bark except some of endophytic isolates; the insects make a tunnel inside the pine tree during the winter and form a chamber around their bodies for protection [31]. When the adults leave the overwintering pine tree logs to feed on fresh leaves, they make contact with the pine bark and thus are exposed to the fungus at that time. That is the starting point of the insecticidal mechanism of the fungus against *M*. *alternatus* because after an adult emerges from the log, it feeds at the bark, allowing fungal contact to be maintained continuously and produce infection. To use the life cycle of *M*. *alternatus* as a control mechanism, it is essential for the fungus to colonize the pine bark and maintain its activity over the winter. To test whether the fungus could be used in the field, we evaluated its insecticidal activity against *M*. *alternatus* when the fungus was treated to the pine bark before emergence, its colonization on the surface of the pine bark, and the control efficacy of our formulated fungal powder in semi-field and field conditions. In addition, we considered the stability of forest biota by evaluating the possibility that infected *M*. *alternatus* could transmit the fungus to non-target forest insects. The results of this study demonstrate that *B*. *bassiana* ERL836 can be used as an environment-friendly agent to effectively control the pine wilt vector *M*. *alternatus*.

*M*. *alternatus* feeding on pines pre-treated with the fungus had a mortality of 70% in 25 days after treatment at 1.0 × 10^7^conidia/ml, and the insecticidal activity was delayed. The high humidity condition was not maintained in this experiment because the pine growing small pots were not sealed; there was only an installed 200 mesh net only to prevent the *M*. *alternatus* adults from escaping from the individual pot. However, we did confirm that *M*. *alternatus* could be killed by the fungus remaining on the pine tree, which can be expected to have a long-term insecticidal activity against pests that attack the tree. We found that the higher the concentration of conidia, the higher the insecticidal activity, so if fungal conidia were treated to living pine trees, the insecticidal activity could be expected to be dose-dependent. In other words, even if the fungal conidia are not treated directly to *M*. *alternatus*, the fungi remaining in the pine trees could have insecticidal effects on pests. However, such a treatment method would require insecticidal tests against non-target insects because fungi can affect other nearby insects when it is treated to pine trees. The application of fungal conidia in forests could adversely affect the ecosystem if it affected non-target insects, so it is difficult to apply in forests below the economic control level, except in areas where PWN is a serious problem. Among the methods for using entomopathogenic fungi to control *M*. *alternatus*, we focused on strategies that treat the fungus to piled pine logs already known to be infected with PWN and insect vectors. Using the management strategies we tested, even a high concentration of insecticide sprayed in a sealed state within a certain area has relatively few adverse effects on the surrounding environment, and high control efficacy can be expected. Previously, we conducted a control study by applying the conidia of a *M*. *anisopliae* isolate on piled wintering pine logs [20]. We confirmed that the fungus colonized the pine tree bark, which suggested the possibility of controlling *M*. *alternatus* by treating overwintering logs with the fungus.

When *M*. *alternatus* adults emerged from inside a tree treated with the *B*. *bassiana* ERL836 conidia suspension, they ate the fungal-treated pine bark, which put them into continuous contact with the fungus. In field conditions, we predicted that the frequency of *M*. *alternatus* adults piercing the covering film and escaping into the surrounding forest would be small. In addition, the conditions for sealing larvae-infested pine logs with a covering film can maintain the relative humidity inside in a saturated state, thereby providing conditions favorable to the growth of fungi. *B*. *bassiana* ERL836 had high insecticidal activity against *M*. *alternatus* adults in the sealed condition, which demonstrates that the activity of the fungus and its insecticidal activity are maintained in high humidity.

*B*. *bassiana* ERL836 was observed to grow on the bark of pine trees (*P*. *koraiensis*, *P*. *densiflora*, and *P*. *thunbergia*) and produced fungal conidia. We observed that mycelium and conidia were generated on the bark of trees treated with the *B*. *bassiana* ERL836-egfp strain, and fluorescence was observed in all the places the fungus grew. On the bark treated with *B*. *bassiana* ERL836, the fungus grew and produced conidia, but no fluorescence was observed, and no fluorescence was observed in the non-treatment group. Therefore, *B*. *bassiana* ERL836 grew on the bark, as suggested not only by the fungal conidia on the treated surface of the pine bark but also by the fungi that colonized the bark and produced additional conidia, which is expected to positively affect the insecticidal activity for a long time.

*B*. *bassiana* ERL836 is a commercialized strain already being used to control thrips. In this study, this strain had high insecticidal activity against *M*. *alternatus*. This commercialized strain can be applied in various forms depending on the stability and use of the fungus. In this study, we devised an application method to replace chemical fumigants by applying the fungus to overwintering pine tree logs known to be infested by *M*. *alternatus*. We evaluated our method in a greenhouse, semi-field, and field conditions: we treated our powder formulation itself by powdering and diluted the powder in water for spray. In the greenhouse conditions, all treatments showed a low emergence rate of *M*. *alternatus* adults, which might have been due to the relatively low temperature and daily temperature fluctuations in the greenhouse (8.5–28.2°C, average 16.3°C.). However, the insecticidal activity of the fungus was maintained, and dead *M*. *alternatus* were observed in the fungal treatment groups. After the fungal suspension spray, the adults died 6–11 days after emergence, and on the 30th day, the percentages of dead individuals among the emerging adults were 73.8% and 82.1% with the 100× and 10× fungal suspension sprays, respectively. On the other hand, after the fungal powder treatment, the adults died 11–12 days after emergence, and on the 30th day, the percentages of dead individuals among the emerging adults were 46.% and 93.3% with the 1 g/box and 10 g/box fungal powder treatments, respectively. Based on the 1 g fungal treatments (both 1 g/box powder and 100× suspension spray using 1 g), the fungal suspension spray had higher insecticidal activity than the powdering, probably because the fungal conidia diluted and suspended in water were treated and colonized the bark evenly, which gave a high probability that the emerging adults would contact the fungal conidia. On the other hand, the powdering showed relatively low insecticidal activity because it could not completely cover the trees even if it was treated evenly across the bark.

However, the 10 g/box powderings covered a larger area than the 1 g/box powdering, increasing both the chance that an adult would contact it and the amount of fungal conidia that attached to the adults and producing insecticidal activity similar to that of the 100× fungal suspension spray. Therefore, control efficacy using the powder form requires enough fungal powder to adequately cover the pine logs within the treated compartment. Thanks to the sealed characteristics of our control strategy, it does not have a significant adverse effect on the environment, even if the fungus is treated at a high concentration. Therefore, the fungal powdering, which is more convenient to use than the suspension spray, seemed suitable for controlling *M*. *alternatus* in a big forest situation. To test that possibility, we evaluated the control efficacy against *M*. *alternatus* in semi-field and field conditions using the formulated fungal powder with the powdering application method. The suspension spray method has a fatal weakness in that it requires water to be moved to forest areas. Thus, a vehicle is required to move a large amount of water to a forested area, making it difficult to apply a suspension in an area where suitable transportation is unavailable. On the other hand, the fungal powder method for treating *M*. *alternatus* larvae–infested pine is a simple matter of carrying the needed amount of powder and then scattering it.

The environmental conditions in which the fungus is applied require a formulation stable in both winter and summer. In fungal biopesticides, the temperature is an important factor. The fungus needs to survive on the pine bark during the low temperatures of winter, and needs to be active when the adults emerge from the pine tree in the spring. The *B*. *bassiana* ERL836 was able to maintain conidia activity for more than two years at a temperature of 4–30°C in previous studies [32]. In addition, it has been reported that *B*. *bassiana* isolates survive at even the low temperature of -15°C and continue to have insecticidal activity when the temperature becomes suitable for growth [33]. The average winter temperature from 2001 to 2020 in Korea was -1.0 ~ 3.1°C, with a minimum of -12.6°C (Statistics Korea, 2021), so fungal conidia can survive the winter. An additional effect of protecting fungal conidia against cryogenic temperatures can be expected by covering the fungal-treated pine logs with the covering film, tarpaulin. The conidia activity of *B*. *bassiana* ERL836 can thus be maintained until the spring of the following year, when the *M*. *alternatus* adults emerge, come into contact with it, are infected, and die. The ERL836 can maintain long-term activity at a temperature of up to 30°C, maintain a conidial germination rate of more than 80% even when stored at 30°C for 18 months, and maintain a high level of insecticidal activity on insects. In other *B*. *bassiana* strains, fungal activity was maintained up to 34°C, but at temperatures higher than that, the activity decreased sharply, making insecticidal activity unlikely [18, 32]. Maintaining insecticidal activity for two years is an important factor in controlling *M*. *alternatus* because the wintering period varies depending on the stage of the larvae. During the wintering period, the larvae of the fourth and fifth instars emerge as adults the following year, but the first–third instars do not emerge until the spring after two winters [34]. In other words, to completely control *M*. *alternatus*, the insecticidal activity of the fungus must be maintained for close to two years. The *B*. *bassiana* ERL836 can be maintained for a long time, so it has a great advantage in controlling *M*. *alternatus*. However, because large temperature changes can greatly reduce the stability of fungi, the stability of *B*. *bassiana* ERL836 should be evaluated under alternating temperature conditions.

Forest environments are more diverse in their biota than agricultural environments. It is difficult to selectively show non-pathogenicity to insects due to the characteristics of fungi, so it is important to seal fungal treatments in specific areas when using them in forest environments. However, *M*. *alternatus* adults could plausibly escape the covering film securing the fungus-treated pine log pile, so whether fungus-infected *M*. *alternatus* adults can transmit the fungus to non-target insects is a major factor in its suitability for use as a biological pesticide in forests. In our results, ERL836 had low virulence in Japanese rhinoceros beetle (*A*. *dichotoma*) adults and Korean stag beetle (*L*. *maculifemoratus*) adults, but high virulence in silkworm (*B*. *mori*) larvae. The results of directly spraying a high concentration of the fungus indicated low virulence against two coleopteran forest insects except silkworm, and the transmission of fungus by fungus-treated adult *M*. *alternatus* was deemed unlikely in those two species. In the case of the silkworm larvae, the survival rate was low even in the non-treated control group when kept with non-infecting *M*. *alternatus* adults, so other factors, such as stress, are the likely cause, rather than the fungus. In the evaluation of transmission potential, the *M*. *alternatus* adults also had a low survival rate, even in the non-treated control group, so stress or fighting with other insects in a confined space is deemed to be the cause of death rather than fungal infection. In a previous experiment, we observed that when adults of the same species were placed in a small space, antennae and other body parts were damaged by fighting with each other (data not shown). The results of the transmission evaluation thus indicate that the impact on the environment from this control strategy is low because ERL836 has relatively low virulence against non-target insects in the forest although the tested insect number was small, and the infected *M*. *alternatus* adults rarely transmit the fungus to other species of insects in close quarters, and the possibility of contact with one another in forest conditions is low due to the covering film.

**Fig 8:**
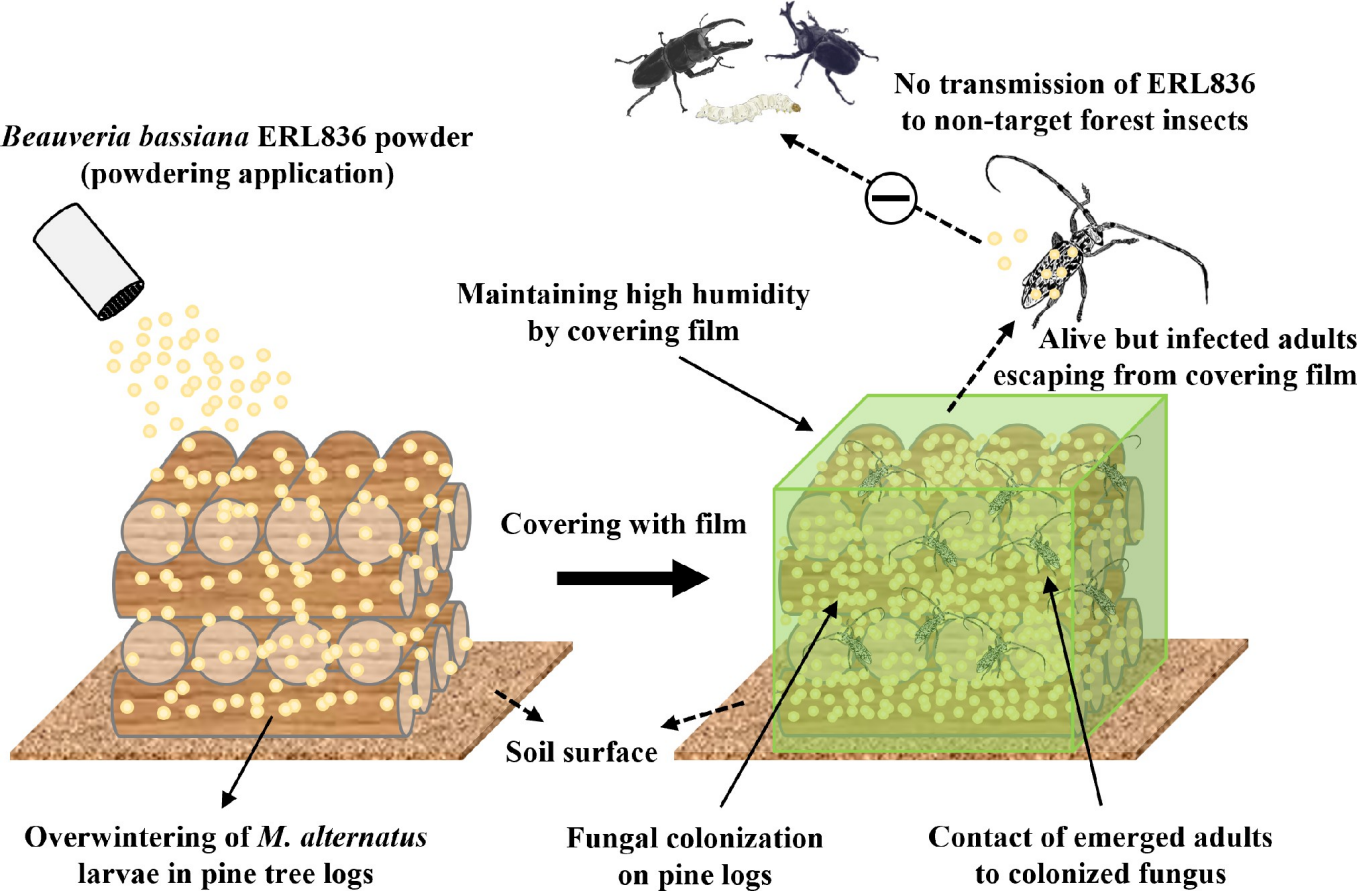
A schematic diagram for controlling *M*. *alternatus* by powdering overwintering pine logs with *B*. *bassiana* ERL836 fungal powder formulation. *B*. *bassiana* ERL836 can be used as a control for *M*. *alternatus* instead of chemical fumigants by simply scattering the fungal powder on pine logs and then covering them with film. The fungus can grow within the film, so it can colonize the surface of the pine logs during overwintering and spring time, and the colonized fungus has insecticidal activity against emerging *M*. *alternatus* adults. If *M*. *alternatus* adults infected with the fungus escape from the film, they are unlikely to transmit the fungus to other non–target insects in the forest, making it suitable for use as an eco–friendly control material without serious adverse effects on forest environment.

Finally, we proposed a strategy to control *M*. *alternatus* by pre-treating the overwintering larvae-infested pine logs with the fungus *B*. *bassiana* ERL836 (**Fig 8**). That strategy can be easily carried out using the formulated fungal powder, and the treated fungus can colonize the pine bark and have insecticidal activity limited to *M*. *alternatus* within the treatment area. In addition, because the control strategy calls for sealing the treated logs, it has the advantage of being able to ensure high humidity and temperature, which maximizes both fungal activity and its expected insecticidal activity against adult insects. We confirmed that the possibility of killing other insects is low even if an infected *M*. *alternatus* adult is exposed to the forest, and we verified that the fungus can be used as a high-efficacy and eco-friendly control agent in forests. However, we can consider one more thing to use entomopathogenic fungi as biopesticides. Although it has been proven that the control efficacy against pest using entomopathogenic fungi through this strategy is high, economic feasibility must be obtained to use formulated entomopathogenic fungi. Fortunately, the entomopathogenic fungus *B*. *bassiana* ERL836 used in this study are already commercialized strains, and it is proved that they have been completed to a considerable degree in culture and formulation. However, in terms of control efficacy and application, it is necessary to compare with chemical agents used previously, and by comparing them, it is proved that the economic value of control using biological pesticides can be improved.

Pine wilt disease, which is caused by pine wilt nematodes, is a major problem in Asia, Europe, and other regions. Eco-friendly materials are needed to replace the chemical fumigation agents currently used to control overwintering insect vectors that mediate pine wilt nematodes. In this study, we used an entomopathogenic fungus, *Beauveria bassiana* ERL836, to replace the previously used chemical fumigant-based control method on pine trees where *Monochamus alternatus* had been found during overwintering. *B*. *bassiana* ERL836 had great insecticidal activity against *M*. *alternatus* adults, and excellent colonization on pine tree surfaces by generating mycelium and conidia. The fungus can withstand low winter temperatures on pine tree bark and act as a deadly pathogen to *M*. *alternatus* adults that emerge from the tree logs after overwintering. A formulated *B*. *bassiana* ERL836 fungal powder treatment on the tree log surfaces is an effective and efficient control strategy, although both of powder and powder-based suspension treatments showed similar insecticidal activity. Rather than diluting the fungal powder in water for sprayable suspension, fungal powder applications do not require equipment beyond a container in which to keep the preparation. Treating the fungal powder to piled and damaged pine tree logs had a low probability of transmitting the fungus to other forest insects, which is an important advantage; entomopathogenic fungi can preserve forest biodiversity except for the target pest. Therefore, the fungal powder preparation has high value as an eco-friendly control strategy that can effectively protect the forest environment. The results presented here could make a great contribution to the eco-friendly management of pine trees by enabling the application of entomopathogenic fungi to major pine forests to control pine wilt nematode vectors.

## Conclusions

Pine wilt disease, which is caused by pine wilt nematodes, is a major problem in Asia, Europe, and other regions. Eco-friendly materials are needed to replace the chemical fumigation agents currently used to control overwintering insect vectors that mediate pine wilt nematodes. In this study, we used an entomopathogenic fungus, *Beauveria bassiana* ERL836, to replace the previously used chemical fumigant-based control method on pine trees where *Monochamus alternatus* had been found during overwintering. *B*. *bassiana* ERL836 had great insecticidal activity against *M*. *alternatus* adults, and excellent colonization on pine tree surfaces by generating mycelium and conidia. The fungus can withstand low winter temperatures on pine tree bark and act as a deadly pathogen to *M*. *alternatus* adults that emerge from the tree logs after overwintering. A formulated *B*. *bassiana* ERL836 fungal powder treatment on the tree log surfaces is an effective and efficient control strategy, although both powder and powder-based suspension treatments showed similar insecticidal activity. Rather than diluting the fungal powder in water for sprayable suspension, fungal powder applications do not require equipment beyond a container in which to keep the preparation. Treating the fungal powder to piled and damaged pine tree logs had a low probability of transmitting the fungus to other forest insects, which is an important advantage; entomopathogenic fungi can preserve forest biodiversity except for the target pest. Therefore, the fungal powder preparation has high value as an eco-friendly control strategy that can effectively protect the forest environment. The results presented here could make a great contribution to the eco-friendly management of pine trees by enabling the application of entomopathogenic fungi to major pine forests to control pine wilt nematode vectors.

## Supporting information

S1 Fig The semi-field and field test to evaluate the control efficacy of *B. bassiana* ERL836 fungal powder against *M. alternatus* by pre-treating the fungus on larvae-infested pine tree logs of *M. alternatus*. (a), Semi-field test on Farmhannong’s test field in Nonsan, Korea. (b), Field test in Ulsan, Korea.
(PDF)
