# Supplementary material for: Management of overwintering pine sawyer beetle, Monochamus alternatus with colonized Beauveria bassiana ERL836
Source: PLoS One. 2022 Sep 2;17(9):e0274086. doi: 10.1371/journal.pone.0274086 (PMC9439257; doi:10.1371/journal.pone.0274086)
Supplement: S1 Fig — (a), Semi-field test on Farmhannong’s test field in Nonsan, Korea. (b), Field test in Ulsan, Korea. (PDF) [file pone.0274086.s001.pdf]

(a) Semi-field trial (Nonsan city)

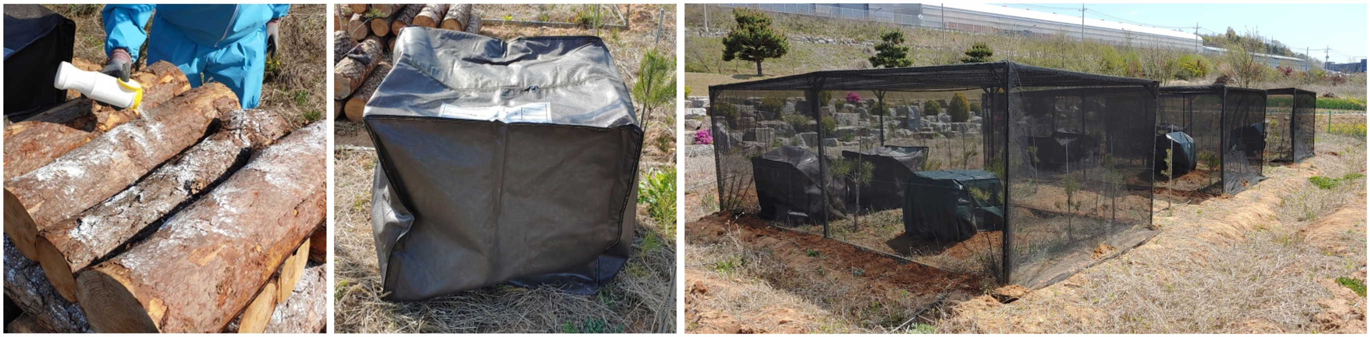

Bb ERL836 powdering on pine bark

Covering a pile of fungal treated pine logs with film

Design of plots in the test semi-field (Grass area of Nonsan city)

(b) Field trail (Ulsan city)

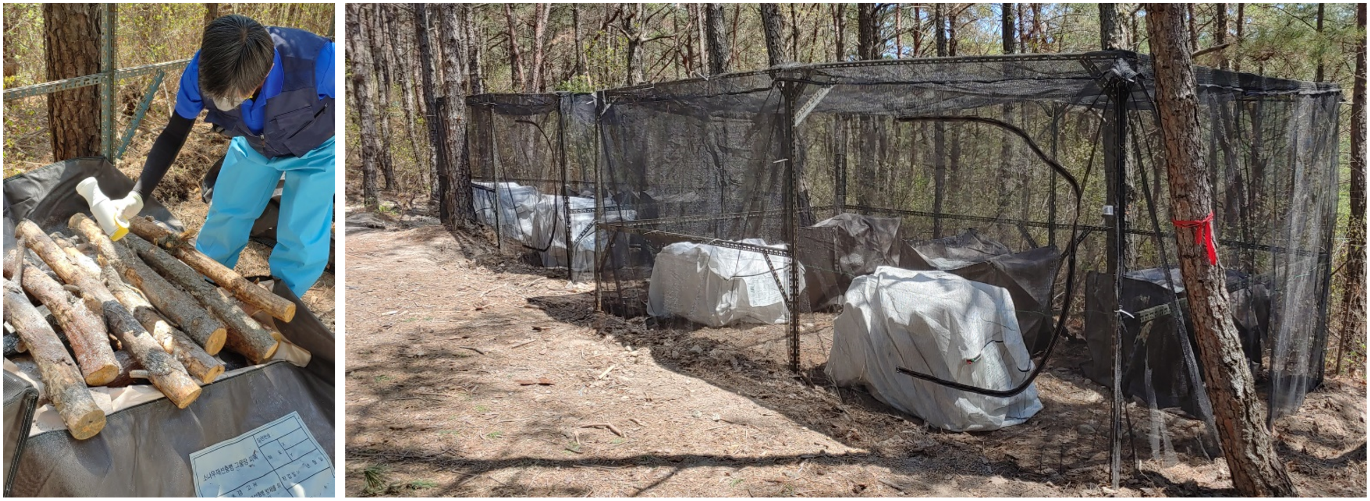

Bb ERL836 powdering on pine bark

Design of plots in the test field (Forest area of Ulsan city)

S1 Fig. The semi-field and field test to evaluate the control efficacy of *B. bassiana* ERL836 fungal powder against *M. alternatus* by pre-treating the fungus on larvae-infested pine tree logs of *M. alternatus*. (a), Semi-field test on Farmhannong's test field in Nonsan, Korea. (b), Field test in Ulsan, Korea.
